# Intrinsically disordered regions in the rodent hepevirus proteome

**DOI:** 10.6026/97320630018111

**Published:** 2022-02-28

**Authors:** Zoya Shafat, Anwar Ahmed, Mohammad K Parvez, Asimul Islam, Shama Parveen

**Affiliations:** 1 Centre for Interdisciplinary Research in Basic Sciences, Jamia Millia Islamia, New Delhi, India; 2 Centre of Excellence in Biotechnology Research, College of Science, King Saud University, Riyadh, Saudi Arabia; 3 Department of Pharmacognosy, College of Pharmacy, King Saud University, Riyadh, Saudi Arabia

**Keywords:** Rat hepevirus, open reading frame (ORF), intrinsic disorder, structured protein, moderately disordered protein, highly disordered protein, ordered protein, intrinsically disordered protein (IDP), intrinsically disordered protein region (IDPR)

## Abstract

Hepatitis E virus (HEV) is the causative agent of Hepatitis E infections across the world. Intrinsically disordered protein regions (IDPRs) or intrinsically disordered proteins (IDPs) are regions or proteins that are characterized by lack of definite structure. These IDPRs or IDPs play significant roles in a wide range of biological processes, such as cell cycle regulation, control of signaling pathways, etc. IDPR/IDP in proteins is associated with the virus's pathogenicity and infectivity. The prevalence of IDPR/IDP in rat HEV proteome remains undetermined. Hence, we examined the unstructured/disordered regions of the open reading frame (ORF) encoded proteins of rat HEV by analyzing the prevalence of intrinsic disorder. The intrinsic disorder propensity analysis showed that the different ORF proteins consisted of varying fraction of intrinsic disorder. The protein ORF3 was identified with maximum propensity for intrinsic disorder while the ORF6 protein had the least fraction of intrinsic disorder. The analysis revealed ORF6 as a structured protein (ORDP); ORF1 and ORF4 as moderately disordered proteins (IDPRs); and ORF3 and ORF5 as highly disordered proteins (IDPs). The protein ORF2 was found to be moderately as well as highly disordered using different predictors, thus, was categorized into both IDPR and IDP. Such disordered regions have important roles in pathogenesis and replication of viruses.

## Background:

Hepatitis E is inflammation of the liver which is caused by the Hepatitis E virus (HEV) [1]. Worldwide, about 20 million HEV infections and 3.3 million symptomatic hepatitis E cases occur annually which results in 44,000 deaths [2]. HEV is of the family Hepeviridae and belongs to the genus Orthohepevirus [3]. The genome of HEV is a single stranded positive-sense RNA (7.2 kb in length), which is flanked with short 5' and 3' non-coding regions (NCR) [4]. The genome comprises three open reading frames (ORFs): ORF1, ORF2 and ORF3. The ORF1, ORF2 and ORF3 encode the non-structural polyprotein (pORF1), capsid protein (pORF2) and the pleotropic protein (pORF3) respectively [5]. Recent studies have determined the role of different reading frame encoded proteins in HEV regulation by analyzing their intrinsically disordered regions [6-9-check with author], as these regions are linked with virus’s infection and pathogenesis [10-12]. The proteins or protein regions that fail to get folded into definite three-dimensional(3D) structures but remain biologically active are termed as intrinsically disordered proteins (IDPs) and intrinsically disordered protein regions (IDPRs), respectively [13-15]. These disordered protein regions exist as extremely active ensembles that are rapidly interconvertible under different physiological conditions [13-15]. Due to occurrence of peculiar phenomenon, i.e., binding of several disordered regions to one ligand or vice-versa (one disordered region binds to many partners), the intrinsic disordered regions are utilized in protein-protein interactions [16, 17]. Therefore, it is of interest to document the intrinsically disordered regions in the rodent hepevirus proteome.

## Materials and methods:

### Sequence retrieval:

The protein sequence (Accession ID: GU345042) of rat HEV was obtained from the NCBI (National Center for Biotechnology Information) GenBank database.

### Structural analysis:

The three-dimensional (3D) model of the rat HEV proteins was modeled using Phyre2 (Protein Homology/AnalogY Recognition Engine) server (http://www.sbg.bio.ic.ac.uk/∼phyre2/html /page.cgi ?id=index) and analyzed.

### Intrinsic disorder prediction:

Intrinsically disordered regions (IDRs) of the rat HEV proteome were predicted using the PONDR® (Predictor of Natural Disordered Regions) (www.pondr.com) at its default settings. Multiple predictors such as members of the PONDR® family including PONDR®VLS2, PONDR®VL3 and PONDR® VLXT were exploited to predict the intrinsic disorder predisposition in rat HEVs. This bioinformatics tool predicts the residues or regions which fail in propensity for an ordered structure formation. The protein residues with predicted scores between 0.2 and 0.5 were considered as flexible, while the residues which had scores, exceeding the 0.5 threshold value, were predicted as intrinsically disordered ones.

## Results and Discussion:

Intrinsic disorder is linked with the pathogenesis and infection of the viruses [10-12]. To complete the life cycle, viruses require various interactions with the components of the host cells. Beginning from the virus's attachment, its entry, commandeering the host machinery, synthesis of the viral components and particle assembly to the last phase, i.e., exiting as new infectious particles from the host cell [18]. All these stages rely heavily on the intrinsic disorder prevalent in viral proteins [18]. The biology of the unstructured regions of the Norway rat HEV, comprising additional reading frames (ORF1, ORF2, ORF3, ORF4, ORF5 and ORF6) remains to be explored. Therefore, the present study reports the analysis on the unstructured regions of the ORF encoded proteins of rat hepevirus to shed novel light on its functionality in HEV regulation. Analysis of protein structure provides a detailed understanding of its function. Thus, the rat HEV protein structures were examined using a web portal for protein modeling and analysis. The modeled 3D structures of rat HEV proteins showed all the three secondary structure states, i.e., alpha helix, beta strand and loops/coils. A study has suggested that loops/coils are not necessarily disordered, however protein disorder is only found within loops [19]. The predicted percentage of disordered residues in the generated 3D rat HEV proteins are summarized in Table 1(see PDF).

Therefore, the initial structural analysis revealed that all the rat HEV proteins consisted of disordered regions (Figure 1A - F - see PDF). The specific role of disordered regions in several nonstructural proteins has been demonstrated to participate in the multiplication and regulatory functions of viruses [20]. For instance, a recent study has shown the involvement of ORF4 protein in the regulation and pathogenesis of HEV due to the presence of significant fraction of disordered regions [21 - check with author]. The disordered regions in the ORF1 Y-domain of HEV have also been shown to perform crucial role in its pathogenesis due to its intrinsic disorder phenomenon [22]. In HDV (hepatitis delta virus), the translation of a delta antigen (a single basic protein) forms the basis of its replication, which is considered as an IDP molecule [23], via both experimental and computational studies [24]. The HCV (Hepatitis C virus) interacts with several viral and host proteins required for its replication via its disordered nonstructural NS5A protein domain [25, 26]. These protein-protein interactions result in the occurrence of several significant biological functions. Moreover, the PPR (Polyproline region) of nonstructural ORF1 has been associated with the regulation of HEV in addition to its role in replication, due to its characteristic intrinsic disorder property [27]. After the initial structural exploration, the intrinsic disorder propensity analysis of rat HEV proteins was carried out to elucidate their intrinsic disorder properties. The predicted intrinsic disordered residues obtained from three disorder predictors for ORF encoded proteins of rat HEV are mentioned in Table 2(see PDF). The resulting disorder profiles of the rat HEV proteins are shown in Figure 2A - F(see PDF).

The rat HEV proteins were initially categorized on the basis of overall degree of intrinsic disorder. The categories included structured proteins (0 - 10%), moderately disordered proteins (10 - 30%) and highly disordered proteins (30 - 100%) [28-29]. Additionally, the ORF proteins were categorized on the basis of length of disordered domains and overall fraction of disordered residues. The categories consisted of ordered proteins (ORDPs); intrinsically disordered protein regions (IDPRs) and intrinsically disordered proteins (IDPs) [28-30]. ORDPs are intrinsic disorder protein variants which consist of less than 30% of disordered residues without disordered domain (consecutive disordered residues) at either C- or N-terminus or in positions distinct from the N- and C-terminals. IDPRs are intrinsic disorder protein variants which consist of less than 30% of disordered residues with disordered domain at either C- or N-terminus or in positions distinct from the N- and C-terminals. IDPs are intrinsic disorder protein variants which consist of more than 30% of disordered residues. Thus, based on these criteria, the obtained disorder profiles of different rat HEV ORF proteins from different disorder predictors are discussed below.

ORF1: The intrinsic disorder profile of the ORF1 protein showed it as a moderately disordered protein, as it consisted of less than 30% (VLXT, VSL2 and VL3) of the disordered residues in its polypeptide chain with two stretches of disordered domains at positions distinct from N- and C-terminals. Thus, were categorized into IDPRs, i.e., structured proteins with intrinsically disordered segments or proteins possessing both structured unstructured regions (Table 2 - see PDF).

ORF2: The intrinsic disorder profile of the ORF2 protein showed it as a highly disordered protein, as it consisted of >30% (as predicted by 40.99% VLXT and 33.07% VSL2) and moderately disordered (17.39% by VL3) as it consisted of less than 30% of the disordered residues in its polypeptide chain along with presence of disordered domain. Thus, on combining these assumptions it was categorized into both IDPs, i.e., proteins having significant fraction of disordered regions or IDPRs, i.e., structured proteins with intrinsically disordered segments (Table 2 - see PDF).

ORF3: The intrinsic disorder profile of the ORF3 protein showed it as a highly disordered protein, as it consisted of >30% (VLXT, VSL2 and VL3) of the disordered residues in its polypeptide chain. Thus, were categorized into IDPs (Table 2 - see PDF).

ORF4: The intrinsic disorder profile of the ORF4 protein showed it as a moderately disordered protein, as it consisted of less than 30% (VLXT, VSL2 and VL3) of the disordered residues in its polypeptide chain with a stretch of disordered domain at N-terminus. Thus, were categorized into IDPRs, i.e., structured proteins with intrinsically disordered segments (Table 2 - see PDF).

ORF5: The intrinsic disorder profile of the ORF3 protein showed it as a highly disordered protein, as it consisted of >30% (VLXT, VSL2 and VL3) of the disordered residues in its polypeptide chain along with possession of long disordered domain towards C-terminus. Thus, were categorized into IDPs (Table 2 - see PDF).

ORF6: The intrinsic disorder profile of the ORF6 protein showed it as a structured protein, as it consisted of less than 30% (VLXT, VSL2 and VL3) of the disordered residues in its polypeptide chain without the presence of any disordered domain. Thus, were categorized into ORDPs, i.e., proteins possessing significant amount of structure (Table 2 - see PDF).

Thus, our intrinsic disorder propensity analysis revealed ORF6 as a highly structured protein (ORDP); ORF1 and ORF4 as moderately disordered proteins (IDPRs); ORF3 and ORF5 as highly disordered proteins (IDPs); and ORF2 as both moderately disordered protein (IPPR) or highly disordered protein (IDP) (Table 3 - see PDF). Interestingly, the computational analysis of revealed ORF3 protein as the most disordered protein, while ORF6 protein as the most ordered protein in the rat HEV proteome with the remaining proteins as intermediates. In context with this, it is noteworthy to mention that all the three intrinsic disorder variants were found in the rat HEV proteins, i.e., ORDP, IDPR and IDP (Table 3 - see PDF). The "IDPR/IDP" is defined as disordered region in protein or disordered protein. These regions/proteins perform a number of significant roles in a variety of biological processes, such as control of signaling pathways, cell cycle regulation, etc. [11,16, 17 & 31]. It has been suggested that IDPRs/IDPs achieve their signaling cascade by binding to their partners with low affinity and high specificity [32]. Thus, the proteins, such as ORF1, ORF2 and ORF4 can play crucial roles in important biological processes as IDPRs. IDP plays significant role in recognition, signaling, regulation and control of protein-protein interaction (PPI) networks [33]. IDPs form essential components of cellular signaling machinery due to its ability to interact differently which results in different consequences [34]. Moreover, they are characterized by enormous flexibility and random conformation (coiled-like). Thus, these distinctive features enable IDPs to participate in one to many and vice-versa interaction [35].

## Conclusion:

The occurrence of the unstructured regions in rat HEV protein sequences suggested their disorder-based binding tendency. The intrinsic disorder analysis revealed all the three intrinsic disorder variants in the proteome of rat HEV. Further, the ORF3 protein was identified as the most disordered protein and ORF6 protein consisted of least fraction of intrinsic disorder. Thus, the rat HEV proteins could be engaged in diverse and essential biological functions.
